# The Effect of Hand Reflexology Massage on Pain and Fatigue in Patients after Coronary Angiography: A Randomized Controlled Clinical Trial

**DOI:** 10.1155/2020/8386167

**Published:** 2020-08-29

**Authors:** Nahid Rejeh, Seyed Davood Tadrisi, Shahrooz Yazdani, Kiarash Saatchi, Mojtaba Vaismoradi

**Affiliations:** ^1^Department of Nursing, Faculty of Nursing and Midwifery, Shahed University, Tehran, Iran; ^2^Trauma Research Center, Faculty of Nursing, Baqiyatallah University of Medical Sciences, Tehran, Iran; ^3^Cardiovascular Research Center, Alborz University of Medical Sciences, Karaj, Iran; ^4^Iranian Scientific Acupuncture Association, Tehran, Iran; ^5^Faculty of Nursing and Health Sciences, Nord University, 8049 Bodø, Norway

## Abstract

**Introduction:**

Coronary angiography can cause pain and fatigue in patients. Hand reflexology as complementary and integrative care approach has been suggested to help with the reduction of patient's pain and fatigue.

**Aim:**

This study aimed to investigate the effect of hand reflexology on pain and fatigue in patients after coronary angiography.

**Design:**

A randomized controlled clinical trial.

**Methods:**

This study was conducted on 90 patients in an angiography department of a referral hospital in an urban area of Iran. The patients were randomly divided into two interventions (*n* = 45) and control (*n* = 45) groups. Hand reflexology was performed for 20 minutes in the intervention group. Pain and fatigue in the groups were measured immediately, 4 and 6 hours after the intervention.

**Results:**

Statistically significant differences were observed in pain and fatigue scores between the groups after the intervention (*P* = 0.001). The intervention had medium to large effects on the patients' pain and fatigue. Hand reflexology as a nonpharmacological and safe technique can be used by nurses along with other pharmacologic interventions in order to reduce patients' suffering related to invasive procedures. The trial is registered with IRCT20110912007529N17.

## 1. Introduction

Cardiovascular disease (CVD) is the major cause of morbidity and mortality across the world [1]. It has become one of the major health concerns in developing countries [2, 3].

Coronary angiography has been recognized as the best method for the diagnosis of CVD [4]. Since the 1940s, this procedure has been used for understanding anatomy, blood circulation, physiology, and pathology of coronary arteries. Coronary angiography plays an important role in the diagnostic of patients with CVD and is accompanied by a minimum risk [5]. Annually, more than 1 million coronary angiographies are performed in the USA, and more than 70% of hospitals perform this procedure on asymptomatic patients [6, 7]. The most common side effects of coronary angiography are bleeding, hematoma, artery thrombosis, pain, and fatigue [8].

Pain is considered the most important complaint experienced by patients after coronary angiography. While analgesic medicines are prescribed to patients after this invasive procedure, they often report mild to moderate pain [9]. After coronary angiography, patients are asked to stay in bed for 6–24 hours to prevent bleeding, hematoma, and embolism. Staying in bed for 6 hours is intolerable and leads to discomfort, backache, and fatigue in patients [10]. Also, the pain increases the number of breaths and reduces the lung volume, because of initiating stress responses, and finally increases the risk of myocardial ischemia [11, 12]. Enough attention should be paid to older patients' complaints regarding pain and fatigue, because they negatively influence their mental health and wellbeing [13]. Therefore, relieving pain and fatigue after coronary angiography needs appropriate interventions by nurses to prevent their negative effects on patients' recovery and the length of hospital stays [9, 14].

## 2. Background

A great challenge of nursing care is to ensure the patient's comfort and relaxation after invasive and painful procedures [15]. Pharmacologic pain management confers both benefit and harm. Nurses often are concerned with adverse drug reactions and side effects of medications that may compromise the patient's health and wellbeing [16]. Analgesic medicines are accompanied by adverse effects including sedation, emesis, anxiety, agitation, and delirium. They may also lead to the prolongation of the length of hospital stays and an increase of healthcare costs [16–18]. On the other hand, nonpharmacologic methods in the form of complementary and alternative medicines are safer [18]. They are nonexpensive interventions, have a few side effects, and can be used in nursing practice [20, 21].

Reflexology is one of the most common complementary methods, but the mechanism underlying its effect has not been clearly understood. It has been suggested that during reflexology energy circulates in the body through vertical zones from the leg toward the head. Therefore, the application of pressure on a reflective point of an organ can impact all organs, glands, bones, and muscles [22]. Reflexology is a comprehensive health method consisting of the application of pressure on the foot and palm. In terms of lexicon, the reflex is nonvoluntary contraction caused by an external stimulant. However, reflex in reflexology means reflection or mirror picture, which reflects small points as a mirror [22]. Since each body organ has reflections in the foot, palm, and ear, reflexology experts believe that hands and feet are the body's mirror and map of the body embodies in hands and feet. Therefore, certain reflective stimulant influences related organs and systems [23, 24]. All organs and glands in the body are associated with reflective points in feet, hands, and ears. Reflexology creates a feeling of security and safety through the reduction of tension and stress [25]. In fact, the application of deep pressure in certain parts of the body can be used for relieving pain [26].

Various theories have been presented to describe the effect of reflexology and one of them is the stimulation of neural receptors through emphasizing the relationship between the central and peripheral nervous systems [23, 24, 27]. Reflexology has been shown to reduce pain and fatigue in chronic diseases such as in patients with lymphoma [28]. Applying pressure on a certain point on the hand and foot can increase blood circulation, neural impulses, and secretion of endorphin and improve the body function [29, 30]. Researchers have raised theories for explaining biological, physiological, and metaphysical mechanisms of reflexology including the energy channel theory, meridian theory, nerve impulse theory, electromagnetic theory, pain gate control theory, and zone theory [24, 31]. According to the gate control theory by Wall and Melzack in 1965, painlessness is caused by the electrical stimulation of the nerve. Reflexology acts as the transcutaneous nerve stimulation (TENS), which transfers the pain message to the brain and blocks the pain perception path. Also, it is believed that reflexology acts by releasing endorphin and encephalin as natural seducers to resist pain [18, 24]. According to Fitzgerald's reflexology zone theory in 1971, the human body is divided into 10 vertical zones as 5 equal zones in each side of the body from the head to thumb. Therefore, the application of pressure by fingers on each side reduces pain at that side. According to the Chi theory, energy circulates in the body in certain pathways [32]. The attentional models of pain perception describe pain reduction in response to reflexology and due to distraction [22, 33]. Therefore, the positive effects of reflexology are the result of the relationship between patients and the therapist, rather than the characteristics of the intervention [34]. This method is often used for symptomatic treatments along with pharmaceutical treatments [35].

Current studies have shown that reflexology is a noninvasive and safe nursing intervention [18, 36, 37]. Hand reflexology can be used to improve physical and pyschological symptoms in patients with various types of health conditions [38–41], but its effect on patients after coronary angiography has not been studied yet. Therefore, this study was conducted to investigate the effect of hand reflexology on pain and fatigue in patients after coronary angiography.

## 3. Materials and Methods

### 3.1. Design

**Figure 1:**
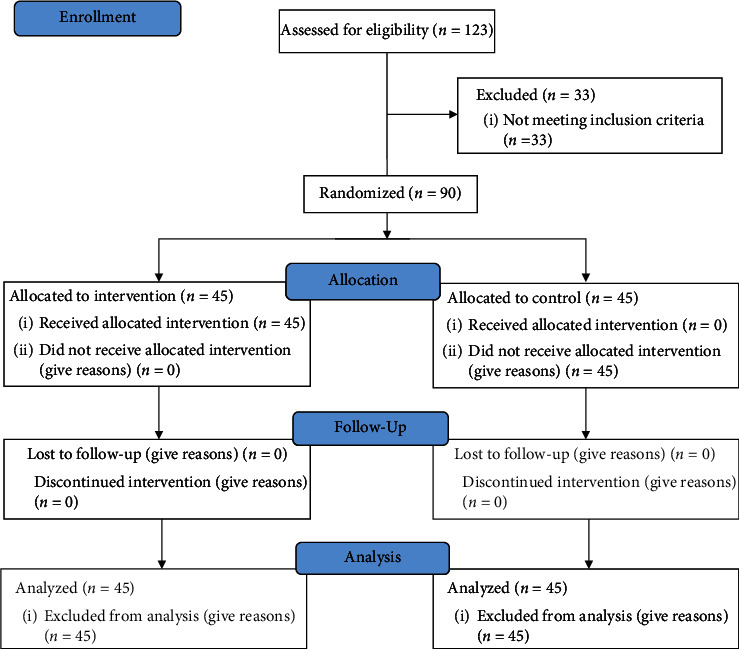
The study process according to the CONSORT flow diagram.

This was a randomized controlled clinical trial with a pre-post intervention design. The outcome measure was the impact of hand reflexology on patients' reported pain and fatigue after coronary angiography.  Figure 1 displays the recruitment, allocation, and follow-up of participants according to the CONSORT flow diagram.

### 3.2. Setting and Sample

This study was conducted on patients scheduled for an elective coronary angiography in a high turnover coronary angiography department of a hospital affiliated with a University of Medical Sciences in an urban area of Iran, from 2 March 2018 to 31 July 2018. This coronary angiography department treats over 280 patients every month.

### 3.3. Eligibility Criteria

The following inclusion criteria were used to select participants: age >18 years, scheduled to undergo coronary angiography for the first time, no invasive procedures such as transesophageal echocardiography prior to coronary angiography, non-emergency coronary angiography, no previous history of coronary angiography, no vascular injuries in the upper limbs and sensory-motor disorders in hands, absence of abnormalities such as corns, burns, amputations, and skin lesions in hands, lack of intervertebral disc herniation, no previous history of mental disorder, and no disturbance in the consciousness level.

Exclusion criteria were hemodynamic instability including dysrhythmia, respiratory disorders, and severe changes in blood pressure that require emergency interventions, bleeding after coronary angiography, and unwillingness to participate in this study.

### 3.4. Sample Size and Recruitment

Given the sample size of a previous study [42], alpha 0.05, 95% confidence interval, power 80%, and 20% possibility of samples' dropout, the sample size was estimated 45 patients in each group using the following sampling formula:

(1) $$\begin{array}{c} n=\frac{{\left(z_{\left(\alpha /2\right)}+z_{\beta }\right)}^{2}*\left(\sigma 1^{2}+\sigma 2^{2}\right)}{d^{2}}=\frac{{\left(1.96+0.85\right)}^{2}*\left(3.05^{2}+2.97^{2}\right)}{{\left(2.68-4.63\right)}^{2}}=37.41≃38. \end{array}$$

Lost = 20% = 7 per group. *α* = 5%, *β* = 20%, *d* = 3.80, *s*1 = 3.05, *s*2 = 2.97, group = 2. Total sample = 2∗45 = 90.

The head nurse of the coronary angiography unit was informed of the study's purpose and inclusion criteria to help with the identification of eligible patients. The patients were selected using a convenient sampling method and were assigned to intervention and control groups using a random sampling method. The group assignments were performed using the block basis sequence by the second author who was unaware of the patients' assignments using a table of random numbers. The random allocation sequence with 23 quadruple blocks with letters A or B indicating the sequence was generated by the statistical adviser. Sealed opaque envelopes containing cards were used, and the size of blocks was not announced to prevent selection bias. The sampling process continued until the required number of the participants was recruited to intervention (hand reflexology) and control (routine nursing care) groups.

### 3.5. Measures

Data was collected using a questionnaire consisting of three parts as follows.

#### 3.5.1. The Demographic Data Form

It was filled out using the patient's medical file or through interviewing the patients. It included items about the patients' age, gender, education level, marital status, employment status, history of smoking or drug use, and medical diagnosis.

#### 3.5.2. Numeric Rating Scale (NRS)

The effect of hand reflexology on self-reported pain was measured using the NSR as a valid instrument for pain assessment in critically ill patients. It is an 11-point scale with equal divisions for self-reporting of pain by adults and children aged 10 years old or older. The range of scores was between 0 and 10 with the following ratings: 0 (lack of pain), 1–3 (mild pain), 4–6 (moderate pain), and 7–10 (severe pain) [43].

#### 3.5.3. The Rhoten Fatigue Scale (RFS)

It consists of a 10 cm line with extremely positive statements on one end and extremely negative statements on the other end. The most positive and negative fatigue statements were scored between 0 and 10, respectively. The ratings of this line were from 0 (lack of fatigue), 1–3 (mild fatigue), 4–6 (moderate fatigue), 7–9 (severe fatigue) to 10 (very severe fatigue). The test-retest method has shown the reliability of this instrument to be 0.93 [44].

### 3.6. Procedure

After obtaining permission to conduct the study, one male nurse and one female nurse received education and training on reflexology for 3 months under the suprevision of a reflexology expert. After 2 hours of coronary angiography, baseline data was collected. The nurses greased the patients' hands using sweet odorless almond oil and performed hand reflexology according to the Ingham's method for 20 minutes as 10-minute pressure on the right hand and then the left hand. First, the whole palm was given the pressure for 2 minutes. Three areas reflecting the solar plexus, heart, and pituitary were pressured. The downward pressure was applied with the thumb in the heart, pituitary, and solar plexus [27, 45]. Next, circular pressure was applied on the same points. The hand reflexology was performed by the male nurse on male patients and by the female nurse on female patients. In the control group, only routine care was provided without hand reflexology consisting of being placed in the supine position in the complete bed rest condition and without receiving any medication. Pain and fatigue levels in the groups were measured immediately after the intervention and 4 and 6 hours after it.

It was impossible to blind the patients with regard to the groups' assignment due to the nature of the intervention. Also, the blindness of the theatre nurses due to the presence of the reflexologist in the unit was impossible. Nevertheless, the statistical analyzer was unaware of the patients' allocation to the groups. In addition, the randomization code was available only to a research fellow who was not connected to this study and was disclosed to the researchers after completing the statistical data analysis.

### 3.7. Statistical Procedures

Descriptive and inferential statistics were used for data analysis via the SPSS software version 20 (SPSS Inc., Chicago, IL, USA). The Kolmogorov-Smirnov test was used to assess data in terms of normal distribution. Data was coded and tabulated to present them in terms of frequency, percentage, mean, and standard deviation. Inferential statistics consisted of the Chi-square test, *t*-tests, and Cramer's V to investigate the intervention's effectivness and its effect size.

### 3.8. Ethical Considerations

The permission to enter the research zone was granted by the ethics committees affiliated with Shahed University (decree code: IR.Shahed.REC. 1396.52). All participants gave their informed consent prior to entering the study. For illiterate patients, the informed consent documentation was read aloud by their companions or relatives and they were asked to add their fingerprints if they were willing to take part in the study. Verbal informed consent was also obtained. Numbers rather than names were used to deidentify the participants and ensure confidentiality and anonymity. The purpose and method of the study were described to the patients. Also, their confidentiality throughout the study was ensured. A cardiologist was available during the procedure to intervene if any adverse effect occurred, but no adverse events or complications related to angiography or hand reflexology were reported by the patients indicating the safety of reflexology. The study research protocol was registered on the Iranian Registry of Clinical Trial under the code of IRCT20110912007529N17.

## 4. Results

Of 123 patients assessed for eligibility, 90 patients met the inclusion criteria and were recruited. All approached agreed to participate and were assigned to either the intervention or the control group (n = 45 in each group). 

### 4.1. Demographic Characteristics of the Participants

The patients in the intervention and control groups had a mean age of 60.60 ± 12.77 years and 57.75 ± 10.34 years, respectively. No statistically significant differences were reported between the groups in terms of sociodemographic and clinical characteristics at the baseline (*P* > 0.05) Table 1.

### 4.2. Pain after the Intervention

At the baseline, the pain level in the control and intervention groups had no statistically significant difference (*P* > 0.05). However, it showed statistically significant differences between the groups during the measurement times (*P*=0.001), and the severity of the effect of reflexology was reported to be large (Table 2).

### 4.3. Fatigue after the Intervention

At the baseline, the fatigue level had no statistically significant difference in the control and intervention (*P* > 0.05). After the intervention, statistically significant differences between the groups during the measurement times were reported (*P* < 0.05). The severity of the effect of reflexology was reported to be moderate immediately after the intervention and was reported large 4 and 6 hours after it (Table 3).

## 5. Discussion

This study aimed to investigate the effect of hand reflexology on the patients' reported pain and fatigue after coronary angiography. After the intervention, it was found that hand reflexology reduced the patients' pain and fatigue. The hand reflexology group reported lower pain and fatigue levels than the control group. While the effect of hand reflexology after coronary angiography has not been studied in the past, our findings concur with those of previous studies indicating the effectiveness of hand reflexology for managing pain or fatigue in patients with various health conditions. Aliasgharpoor et al. and Shaer Moghadam et al. [38, 41] found that hand reflexology reduced fatigue in patients undergoing hemodialysis. Irani et al. [39] showed the effect of hand reflexology on the reduction of postcesarean pain and anxiety. The study by Wang and Keck [46] supported its effect on postoperative pain. Cassileth and Vickers [47] reported substantive improvements in the symptoms of patients with cancer after hand reflexology. Hodgson and Lafferty [48] studied the effects of reflexology and Swedish massage on stress and pain in older cancer survivors in nursing homes. Accordingly, both types of massage improved patients' outcomes. Rambod et al. [28] showed that reflexology was helpful for the reduction of fatigue and pain in patients with lymphoma.

The application of hand reflexology only for one session can be considered a limitation of this study. Therefore, to remove the effect of the “healing crisis” or “cleansing process,” [49] the reflexology intervention should be applied in more sessions. Impossibility to blind the researchers and the participants to the group allocations and the assessment of pain and fatigue using the self-report method are the other limitations of this study.

## 6. Conclusions

Hand reflexology reduced fatigue and pain in patients after coronary angiography. It is a nonexpensive and safe intervention that does not need any special equipment unless specific instructions that are given to nurses on how to implement it and how to incorporate it into routine nursing practice. The use of complementary and integrative medicines as safe and nonpharmacologic interventions for the reduction of pain and fatigue in patients undergoing invasive procedures can improve nurses' independence in decision-making and increase their self-confidence during patient care. Therefore, its education is suggested to be incorporated into academic nursing education and on-the-job training in critical and intensive care units. Further studies are required to investigate the impact of hand reflexology in comparison with other complementary medicines approaches on pain, fatigue, and wellbeing in patients undergoing painful and invasive procedures. Also, the use of a placebo group can enable making firmer conclusions regarding the impact of reflexology on patients' reported pain and fatigue in relation to the attentive presence of the reflexologist. Replicating this research in other healthcare settings and on patients with chronic diseases is suggested.

## Figures and Tables

**Table 1:** The baseline characteristics of the patients in the groups.

Variable		Control (*n* = 45)	Intervention (*n* = 45)	test, *P* value
Age mean (SD)		57.75 (10.34)	60.60 (12.77)	*t* (88) = −1.16, *P*=0.24
Kolmogorov–Smirnov test		D (45) = 0.12, *P* = 0.06	D (45) = 0.06, *P* = 0.20	
Variable	*n* (%)	Control (*n* = 45)	Intervention (*n* = 45)	test, *P* value
Gender	Male	33 (36.7%)	24 (26.7%)	Chi-square *X*^2^ (1) = 3.87, *P*=0.07
	Female	12 (13.3%)	21 (23.3%)	
Married	Married	40 (44.4%)	32 (35.6%)	Chi-square *X*^2^ (1) = 4.44, *P*=0.06
	Single	5 (5.6%)	13 (14.4%)	

Education level	Illiterate	16 (17.7%)	13 (14.4%)	Chi-square *X*^2^ (4) = 4.36, *P*=0.35
	Elementary	8 (8.9%)	12 (13.3%)	
	Middle school	6 (6.7%)	9 (10%)	
	Diploma	4 (4.4%)	6 (6.7%)	
	Academic	11 (12.2%)	8 (8.9%)	

Occupation	Housekeeper	11 (12.2%)	12 (13.3%)	Chi-square *X*^2 (^3) = 0.18, *P*=0.98
	Self-employed	14 (15.6%)	13 (14.4%)	
	Employee	10 (11.1%)	9 (10%)	
	Retired or disabled	10 (11.1%)	11 (12.2%)	

Smoking and drug use	Tobacco	8 (8.9%)	7 (7.8%)	Chi-square, *χ*^2^ (3) = 0.70, *P*=0.87
	Opium	7 (7.8%)	5 (5.6%)	
	Both	6 (6.7%)	8 (8.9%)	
	None	24 (26.7%)	25 (27.8%)	

Primary diagnosis	Coronary disease	15 (16.7%)	13 (14.4%)	Chi-square, *χ*^2^ (3) = 1.38, *P*=0.71
	Myocardial infarction	12 (13.3%)	14 (15.6%)	
	Unstable angina	3 (3.3%)	10 (11.1%)	
	Ventricular disease	5 (5.6%)	8 (8.9%)	

Nitro drip	No	13 (14.4%)	12 (13.3%)	Chi-square, *χ*^2^ (1) = 0.05. *P*=0.99
	Yes	32 (35.66%)	33 (36.7%)	

**Table 2:** Comparison of pain between the groups before the intervention and at follow-up.

Time	Group (*n* = 45)	No (0) *n* (%)	Mild (1–3) *n* (%)	Moderate (4–6) *n* (%)	Severe (7–10) *n* (%)	test, *P*-value
Baseline	Control	—	4 (4.4%)	30 (33.3%)	11 (12.2%)	Chi-square *χ*2 (2) = 4.86.*P*=0.08
	Intervention	—	12 (13.3)	24 (26.7%)	9 (10%)	

Immediately after the intervention	Control	—	5 (5.6%)	13 (14.4%)	27 (30%)	Chi-square *χ*2 (2) = 32.45. *P*=0.001 Cramer's V = 0.60 Large effect
	Intervention	—	24 (26.7%)	18 (20%)	3 (3.3%)	

4 hours after the intervention	Control	0 (0%)	10 (11.1%)	18 (20%)	17 (18.9%)	Chi-square *χ*2 (3) = 35.95. *P*=0.001 Cramer's V = 0.63 Large effect
	Intervention	11 (12.2%)	26 (28.9%)	6 (6.7%)	2 (2.2%)	

6 hours after the intervention	Control	—	3 (3.3%)	15 (16.7%)	27 (30%)	Chi-square *χ*2 (2) = 42.87. *P*=0.001 Cramer's V = 0.69 Large effect
	Intervention	—	31 (34.4%)	11 (12.2%)	3 (3.3%)	

**Table 3:** Comparison of fatigue between the groups before the intervention and at follow-up.

Time	Group (*n* = 45)	No (0)*n* (%)	Mild (1–3) *n* (%)	Moderate (4–6) *n* (%)	Severe (7–9) *n* (%)	Very severe (10) *n* (%)	test, *P* value
Baseline	Control	5 (5.6)	–	16 (17.8)	–	24 (26.7)	Chi-square *χ*2 (2) = 4.58. *P*=0.10
	Intervention	8 (8.9)	–	23 (25.6)	–	14 (15.6)	

Immediately after the intervention	Control	3 (3.3)	5 (5.6)	11 (12.2)	9 (10)	17 (18.9)	Chi-square *χ*2 (4) = 12.48. *P*=0.01 Cramer's V = 0.37 Medium effect
	Intervention	6 (6.7)	10 (11.1%)	19 (21.1)	5 (5.6)	5 (5.6)	

4 hours after the intervention	Control	0 (0)	7 (7.8)	10 (11.1)	24 (26.7)	4 (4.4)	Chi-square *χ*2 (4) = 43.36. *P*=0.001 Cramer's V = 0.69 Large effect
	Intervention	6 (6.7)	28 (31.1)	2 (2.2)	2 (2.2)	7 (7.8)	

6 hours after the intervention	Control	0 (0)	8 (8.9)	13 (14.4)	19 (21.1)	5 (5.6)	Chi-square *χ*2 (4) = 42.70. *P*=0.001 Cramer's V = 0.68 Large effect
	Intervention	6 (6.7)	27 (30)	3 (3.3)	0 (0)	9 (10)	

## Data Availability

Data supporting the results reported in this article can be achieved by sending the request to the corresponding author.
